# Platelet CXCL4 mediates neutrophil extracellular traps formation in ANCA-associated vasculitis

**DOI:** 10.1038/s41598-020-80685-4

**Published:** 2021-01-08

**Authors:** Kotaro Matsumoto, Hidekata Yasuoka, Keiko Yoshimoto, Katsuya Suzuki, Tsutomu Takeuchi

**Affiliations:** 1 Division of Rheumatology, Department of Internal Medicine, Keio University School of Medicine, 35 Shinanomachi, Shinjuku-ku, Tokyo, 160-8582 Japan; 2 Division of Rheumatology, Department of Internal Medicine, Fujita Health University School of Medicine, 1-98 Dengakugakubo, Kutsukake-Cho, Toyoake, Aichi 470-1192 Japan

**Keywords:** Rheumatic diseases, Immunology

## Abstract

Neutrophils form neutrophil extracellular traps (NETs), which are involved in the pathogenesis of ANCA-associated vasculitis (AAV). Recent reports suggest that platelets stimulated via toll-like receptor (TLR) pathways can induce NETs formation. However, the mechanism underlying the involvement of platelets in NETs formation in AAV is unknown. We investigated the role of platelets in the pathogenesis of AAV. Platelets from AAV patients and healthy controls (HCs) were co-cultured with peripheral neutrophils, and NETs formation was visualized and quantified. The expression levels of TLRs on platelets were examined by flow cytometry. Platelets were treated with a TLR agonist, platelet-derived humoral factor, CXCL4 (platelet factor 4: PF4), and/or anti-CXCL4 antibody to investigate the effects of TLR–CXCL4 signaling on NETs formation. Platelets from AAV significantly upregulated NETs formation in vitro. Flow cytometric analysis revealed that the proportion of TLR9 positive platelets was significantly higher in AAV than HCs. CXCL4 released from TLR9 agonist-stimulated platelets was significantly enhanced in AAV, which subsequently increased NETs formation. Further, neutralizing anti-CXCL4 antibody significantly inhibited NETs formation enhanced by platelets from AAV. TLR9 signaling and CXCL4 release underlie the key role that platelets play in NETs formation in the pathogenesis of AAV.

## Introduction

ANCA-associated vasculitis (AAV) is an autoimmune disease that affects small- to medium-sized blood vessels and causes vascular inflammation and multiple organ damage^1–3^. AAV is primarily managed with high-dose glucocorticoid in combination with cyclophosphamide or rituximab^4–6^. Despite improvements following the introduction of cyclophosphamide- or rituximab-based treatments, however, patients often experience disease relapses^5–8^. While the pathogenesis of AAV remains unclear, several reports have shown that vascular endothelial dysfunction due to neutrophil extracellular traps (NETs) may be an important trigger in the disease process^9^.

NETs, chromatin-derived fibers released from neutrophils, exhibit antimicrobial functions by trapping and killing extracellular pathogens in circulation and peripheral tissues^10–12^. While NETs plays a critical role in host defense, excessive formation or persistence of NETs may lead to adverse effects. For example, NETs formation has been commonly observed in connective tissue diseases such as AAV, systemic lupus erythematosus (SLE), rheumatoid arthritis (RA) and systemic sclerosis^9,13–15^. NETs have been confirmed in patients and rodent models of AAV^16,17^. In addition, improvement of vasculitis by NETs suppression has been reported improving vasculitis in mice models^18,19^. Although the regulatory mechanisms of NETs have not been sufficiently clarified, platelets have been reported as one of cellular regulators of NETs^20,21^. Moreover, Clark et al. showed that platelets were involved in NETs formation via toll-like receptor 4 (TLR4) pathways in a mouse model^22^. These studies suggest that TLR pathways in platelets may be involved in the activation and regulation of neutrophils and NETs formation in human disease.

Recent reports have shown that platelet activation contributes to the progression of AAV pathogenesis by triggering the alternative complement pathway or release of proinflammatory microparticles^23,24^. The mechanisms governing the acceleration of NETs formation in AAV are not fully understood. Given recent evidence that TLR signaling pathways may play an important role in the development of chronic inflammation in rheumatic diseases^25^, we hypothesized that activation of neutrophils by TLR signaling-activated platelets may contribute to the acceleration of NETs formation in patients with AAV.

Here, we investigated the role of platelets in the pathogenesis of AAV, particularly in NETs formation, and the involvement of TLR signaling.

## Methods

### Patients and healthy controls

Patients with AAV who visited Keio University Hospital and fulfilled the 2012 Revised International Chapel Hill Consensus Conference Nomenclature for granulomatosis with polyangiitis (GPA) and microscopic polyangiitis (MPA) were consecutively enrolled between August 2017 and January 2020. Patients with SLE and RA^26–28^, and healthy controls (HCs) were also included. HCs were confirmed to have no autoimmune diseases, severe allergic disorders, malignancies or infections.

This study was approved by the research ethics committee of Keio University School of Medicine (#20140335) and was conducted according to the Declaration of Helsinki. Informed consent was obtained from all patients and HCs.

### Clinical assessment

Disease activity of AAV was determined using BVAS together with clinical signs of activity^29^. Clinical information was obtained from patients’ records. Organ involvement of ear, nose, throat (ENT), lung, kidney and CNS, and laboratory parameters such as white blood cell counts, platelet counts, C-reactive protein (CRP), ANCA positivity, ANCA-titer (by chemiluminescent enzyme immunoassay), and Krebs Von Den Lungen-6 (KL-6) in serum were also assessed.

### Isolation of neutrophils and platelets

Neutrophils were isolated from heparinized blood samples using Mono-Poly Resolving Medium (DS Pharma Biomedical, Osaka, Japan) and suspended in culture medium containing DMEM at 8 ℃. The proportion of neutrophils was determined using Wright-Giemsa staining. In order to clarify the role of platelets and platelet-associated humoral factors in the process of NETs formation, neutrophils from HCs, which was not primed or affected by the immunological environment, were selected to use in most of the experiments. To evaluate platelets that were as fresh as possible, we compared the effects between platelet-poor plasma (PPP) and platelet-rich plasma (PRP), according to previously published method^30^. PRP, containing a large number of platelets from the subject's blood, and PPP, the supernatant of PRP were prepared by precipitating platelets by centrifugation at room temperature^31^.

### Analysis of NETs formation

We examined NETs formation visually using a live-cell imaging system as reported previously ^31^. Briefly, neutrophils on a 35-mm glass dish (Matsunami, Tokyo, Japan) were incubated with 500 nM Picogreen (Thermo Fisher Scientific, San Jose, CA, USA) for staining extracellularly-released DNA and 500 nM HySOx (Goryo chemical, Sapporo, Japan) for probing hypochlorous acid. Confocal fluorescence microscopy (FV-10; Olympus, Tokyo, Japan) was performed in a CO_2_ incubator at 37 ˚C and the area of positive signal of Picogreen was quantified using ImageJ processing software (U. S. National Institutes of Health, Bethesda, MD, USA). NETs formation was also quantified by measuring the fluorescence intensity of Picogreen-labeled extracellular DNA from 1 × 10^5^ neutrophils in 300 μL culture medium. In some experiments, anti-MPO (Clone 2C7; BIO-RAD, Hercules, CA, USA) and anti-Histone H3 (Abcam, Cambridge, UK) antibodies were used to stain NETs components.

### Platelet-neutrophil co-culture assay

To examine the effect of platelets on NETs formation, a platelet-neutrophil co-culture system was established. Neutrophils and platelets were prepared and co-cultured in an allogenic manner. The number of platelets was adjusted to 5 × 10^6^ per 10 μL using autologous PPP. Neutrophils were seeded onto 96-well plates (Corning, NY, USA) at 1 × 10^5^ cells per well and incubated with pre-stimulated PPP, PRP or culture supernatant for 1 h. We defined the difference of DNA concentration as platelet-mediated NETs. The difference of DNA concentration was calculated in stimulation with PPP and PRP. A trans-well assay kit (Corning) with 0.4 μm pore size was used to evaluate the effect of platelet adhesion to neutrophils.

To examine the effect of TLR signaling, CpG oligodeoxynucleotides (Novus Biologics, Littleton, CO, USA) as a TLR ligand or control oligodeoxynucleotides (Novus Biologics) were used. In order to evaluate humoral factors released from CpG-stimulated platelets, we need to test under the condition that plasma effect on neutrophils is small. Based on product information and previous report^32^, 5 μL of PRP was stimulated with 50 μL of CpG/CpG control (0, 10, 20 μg/mL) solution for 16–18 h at 37 °C, and their supernatants were used for further analysis. We hypothesized that NETs-DNA stimulates platelets via TLR9, and constructs positive loop to enhance NETs formation in AAV. To test our hypothesis, we isolated NETs-DNA as published previously ^33^ and stimulated platelets with NETs-DNA (20 μg/mL), then measured NETs formation and CXCL4 release. To determine the role of platelet TLRs on NETs formation, the TLR9 inhibitory oligodeoxynucleotide, ODN TTAGGG (A151) (InvivoGen, San Diego, CA, USA) was used.

A recombinant chemokine (C-X-C motif) ligand (CXCL) 4 (platelet factor 4: PF4) protein (PeproTech, Rocky Hill, NJ, USA) was used to ascertain whether CXCL4 directly induced NETs formation. Recombinant CXCL4-mediated NETs formation was assessed by measuring the NETs area and quantifying Picogreen fluorescence intensity. To determine the role of platelet-derived humoral factors on NETs formation, an anti-CXCL4 antibody (US Biological, Salem, MA, USA) was used.

### Flow cytometry

To identify the cellular phenotype of platelets, expression of surface molecules was examined by flow cytometry. For gating purposes, the platelet population was verified using anti-CD41a-VioBlue (Clone REA386; Miltenyi Biotec, Auburn, CA, USA) monoclonal antibody (mAb) staining. Antibodies used to probe surface molecules included anti-P-selectin-FITC (Clone CLBThromb/6; Beckman Coulter, Westbrook, ME, USA), anti-TLR2-Alexa Fluor 488 (Clone T2.5; Abcam), anti-TLR4-APC (Clone HTA125; Abcam) and anti-TLR9-FITC (Clone 5G5; Abcam) mAbs. Isotype-matched control IgGs for each mAb were used to set negative populations. Platelets were analyzed on a MACS Quant Analyzer (Miltenyi Biotec) and data were analyzed using FlowJo v.7.6.4 software (Tree Star, Ashland, OR, USA). Details of the gating strategies of platelets are shown in Fig. S1. As shown in Fig. S1, TLR9 is found in intracellular domains, though it has been reported that TLR9 could migrate to the outer membrane after activation^34^. TLR9 expression was measured after permeabilization of the cells using cytofix/cytoperm permeabilization kit (BD Biosciences, San Jose, CA, USA).

### Measurement of secreted proteins

Level of CXCL4 in plasma and culture supernatant was determined using a human CXCL4 ELISA kit (R&D Systems, Minneapolis, MN, USA) according to the manufacturer’s protocol. Plasma samples and culture supernatants stored at –80 ˚C prior to ELISA were used in the assay.

### Statistical analysis

Continuous data are expressed as mean ± SD, and categorical data as number and/or percentage. The Mann–Whitney *U* test was used to examine differences between 2 groups and the chi-squared test for nominal variables. Multiple comparison was assessed using the Kruskal–Wallis test and post-hoc Mann–Whitney *U* test. Pearson’s correlation coefficient was used for correlation analysis. P values less than 0.05 were considered significant. All analyses were performed using JMP version 13.0 (SAS Institute, Cary, NC, USA) or GraphPad Prism software version 8.0 (GraphPad, La Jolla, CA, USA).

## Results

### Baseline characteristics of patients

Patients with AAV (Total: n = 22, GPA: n = 13, MPA: n = 9), SLE (n = 10), RA (n = 12) and HCs (n = 20) were consecutively enrolled. Patients’ baseline characteristics are summarized (Table 1 and S1).

**Table 1:** Clinical characteristics of patients with AAV.

Parameter	AAV (n = 22)
**Baseline characteristics**	
Age, years	69 ± 17
Male, n (%)	11 (50)
GPA/MPA, n	13/9
BVAS	7.6 ± 7.4
Newly diagnosed/under treatment, n (%)	18 (82)/4 (18)
**Characteristics of patients under treatment**	
Disease duration, months	11 ± 3.5
Prednisolone dose, mg	10 ± 5.4
**Laboratory tests**	
WBC, cells/μL	9300 ± 3937
Neutrophils, cells/μL	7368 ± 3343
Lymphocytes, cells/μL	1250 ± 824
Monocytes, cells/μL	426 ± 253
Eosinophils, cells/μL	211 ± 424
Basophils, cells/μL	41 ± 41
Platelets, × 10^4^ cells/μL	31 ± 14
Creatinine, mg/dL	0.8 ± 0.5
eGFR, mL/min/1.73 m^2^	77 ± 24
CRP, mg/dL	5.2 ± 4.7
MPO-ANCA positive, n (%)	14 (64)
PR3-ANCA positive, n (%)	7 (32)
MPO/PR3-ANCA titer, IU/L	71 ± 89
Rheumatoid factor positive, n (%)	13 (59)
KL-6, U/mL	414 ± 298
**Organ involvement**	
Ear, nose, throat, n (%)	12 (55)
Lung, n (%)	11 (50)
Kidney, n (%)	6 (27)
Central nervous system, n (%)	4 (18)

*AAV* ANCA-associated vasculitis, *GPA* granulomatosis with polyangiitis, *WBC *white blood cell, *KL-6* Krebs Von Den Lungen-6.

All patients with AAV were Japanese, and the median age and male to female ratio was 69 ± 17 years and 1:1 (11:11), respectively. The median BVAS score at baseline was 7.6 ± 7.4 and 95% (21/22) of patients were ANCA positive. Average CRP level and ANCA titer were 5.2 ± 4.7 mg/dL and 71 ± 89 IU/mL. Involved organs included the ENT (55%; 12/22), lung (50%; 11/22), kidney (27%; 6/22) and CNS (18%; 4/22). The higher proportion of patients with lung involvement compared to other organs is consistent with previous epidemiological study from Japan^35^.

### Platelets from AAV patients strongly induced NETs formation

To investigate the effect of platelets from AAV patients on NETs formation, we used a co-culture system comprising neutrophils and PPP or PRP. The difference in stimulation with PPP and PRP was used to define platelet-mediated NETs formation to reduce the effects of platelet activation due to enrichment of the platelet fraction.

**Figure 1:**
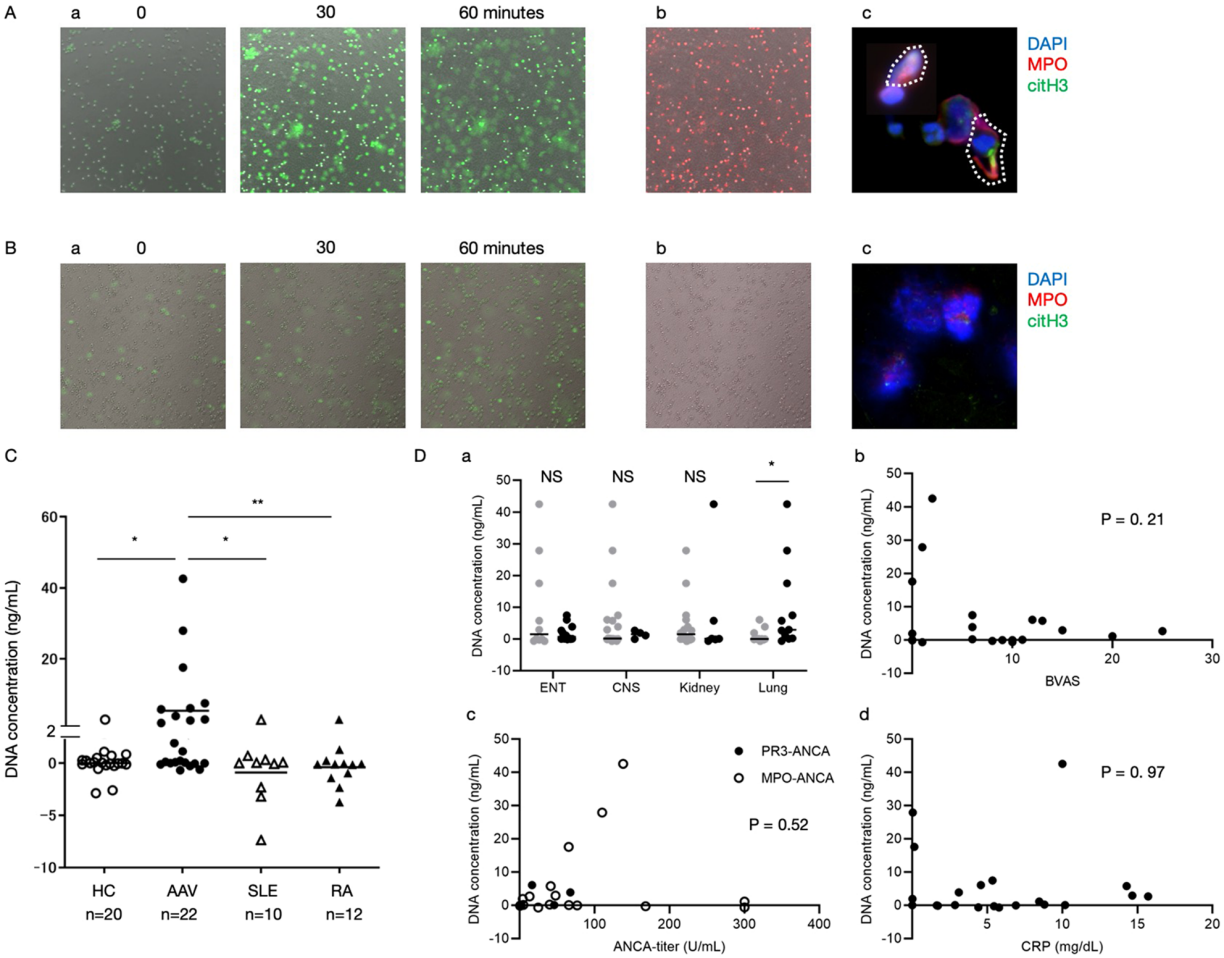
Platelets from AAV patients enhanced NETs formation. Representative images of NETs formation with **(A)** platelet-rich plasma (PRP) and **(B)** platelet-poor plasma (PPP) stimulation. Time-lapse images (0, 30, 60 min) obtained using confocal microscopy of **(a)** extracellular DNA and **(b)** reactive oxygen species. **(c) **Neutrophils immunohistochemically-stained with MPO and citrullinated histone (citH3). Scale bar: 20 μm. **(C) **Concentrations of DNA released from neutrophils from healthy controls (HCs) were measured. The difference of DNA concentration was calculated in stimulation with PPP and PRP from HCs (n = 20) and ANCA-associated vasculitis (AAV) (n = 22), systemic lupus erythematosus (SLE) (n = 10) and rheumatoid arthritis (RA) (n = 12) patients. **(D)** Associations of the level of platelet-mediated NETs with **(a)** organ involvement, **(b)** BVAS, **(c)** ANCA titer and **(d)** CRP. *p < 0.05 **p < 0.01 ***p < 0.001 for analysis using Mann–Whitney *U* test.

Representative time-lapse images obtained using confocal microscopy of extracellular DNA following co-culture of peripheral neutrophils with PPP or PRP from AAV patients are shown (Fig. 1A-a,B-a). Since the generation of reactive oxygen species (ROS) is one of the characteristics for NETosis^36^, hypochlorous acid-labeled ROS production is also shown (Fig. 1A-b,B-b). In addition, neutrophils immunohistochemically-stained with MPO and citrullinated histones are shown (Fig. 1A-c,B-c). PRP from AAV patients significantly enhanced neutrophils from HCs to form NETs.

Representative images showing DNA released from NETs following co-culture of peripheral neutrophils with PPP or PRP from AAV patients or HCs are shown (Fig. S2A). Neutrophils from AAV patients spontaneously induced NETs formation in vitro even in the absence of PPP or PRP stimulation, while neutrophils from HCs did not (a, b). PPP or PRP from HCs (c, d) and AAV patients (e, f) were used to investigate the effect of platelets on neutrophils from HCs promoting NETs formation. Neutrophils from HCs markedly enhanced NETs formation in the presence of platelets from AAV patients (f), whereas platelets from HCs did not induce NETs formation (d). We excluded the transfer with their PPP and PRP pool (g, h). These results show that platelets from AAV patients had NETs formation-promoting effects; however, the mechanism was not clear. To determine whether direct contact between platelets and neutrophils is necessary for activation of neutrophils, we cultured neutrophils and platelets separately using a trans-well insert. As shown in Fig. S2A (i), platelets from AAV patients induced NETs formation in the absence of adhesion to neutrophils. These data suggest that platelets induce NETs formation via not only cell-to-cell contact with neutrophils but also humoral factors produced by platelets.

### Association of platelet-mediated NETs with clinical parameters

Results of the quantification of NETs formation in HCs (n = 20) and patients with AAV (n = 22), SLE (n = 10) and RA (n = 12) are shown (Fig. S2B). Level of NETs formation induced by PRP from AAV patients was significantly higher than PPP from AAV patients, and that induced by PPP from AAV patients was higher than PPP from HCs. Since the effect of PPP from AAV patients was dominantly observed, the difference of DNA concentration in stimulation with PPP and PRP was calculated (Fig. 1C). Kruskal–Wallis test revealed that the levels of NETs formation induced by platelet-fraction were statistically different among HCs, and patients with AAV, SLE and RA (p = 0.015). Post-hoc test revealed that platelet-mediated NETs formation from AAV patients (5.4 ng/mL) was higher than those from HCs (0.0026 ng/mL, p = 0.025), SLE (− 0.90 ng/mL, p = 0.047) and RA patients (-0.37 ng/mL, p = 0.0047).

Comparison among patients with selected organ involvement demonstrated that several patients with MPO-ANCA^+^ MPA who had lung involvement but not ENT and CNS involvement had higher levels of platelet-mediated NETs. Among organ involvements, patients with AAV with lung lesion had significantly higher level of platelet-mediated NETs than in those without (2.9 vs 0.0077 ng/mL, p = 0.020) (Fig. 1D-a), while there was no difference in NETs formation among patients with and without organ involvements with ENT and CNS. Correlation analysis showed no significant association of platelet-mediated NETs with BVAS (Fig. 1D-b), ANCA titer (Fig. 1D-c) or CRP (Fig. 1D-d). Figure 1D-c showed no difference between platelets from PR3- and MPO-ANCA patients. Comparison of the levels of platelet-mediated NETs between treatment naïve and under treatment were not significantly different in patients with SLE and RA (Table S2).

### Elevated TLR9 expression in platelets is correlated with clinical features of AAV patients

**Figure 2:**
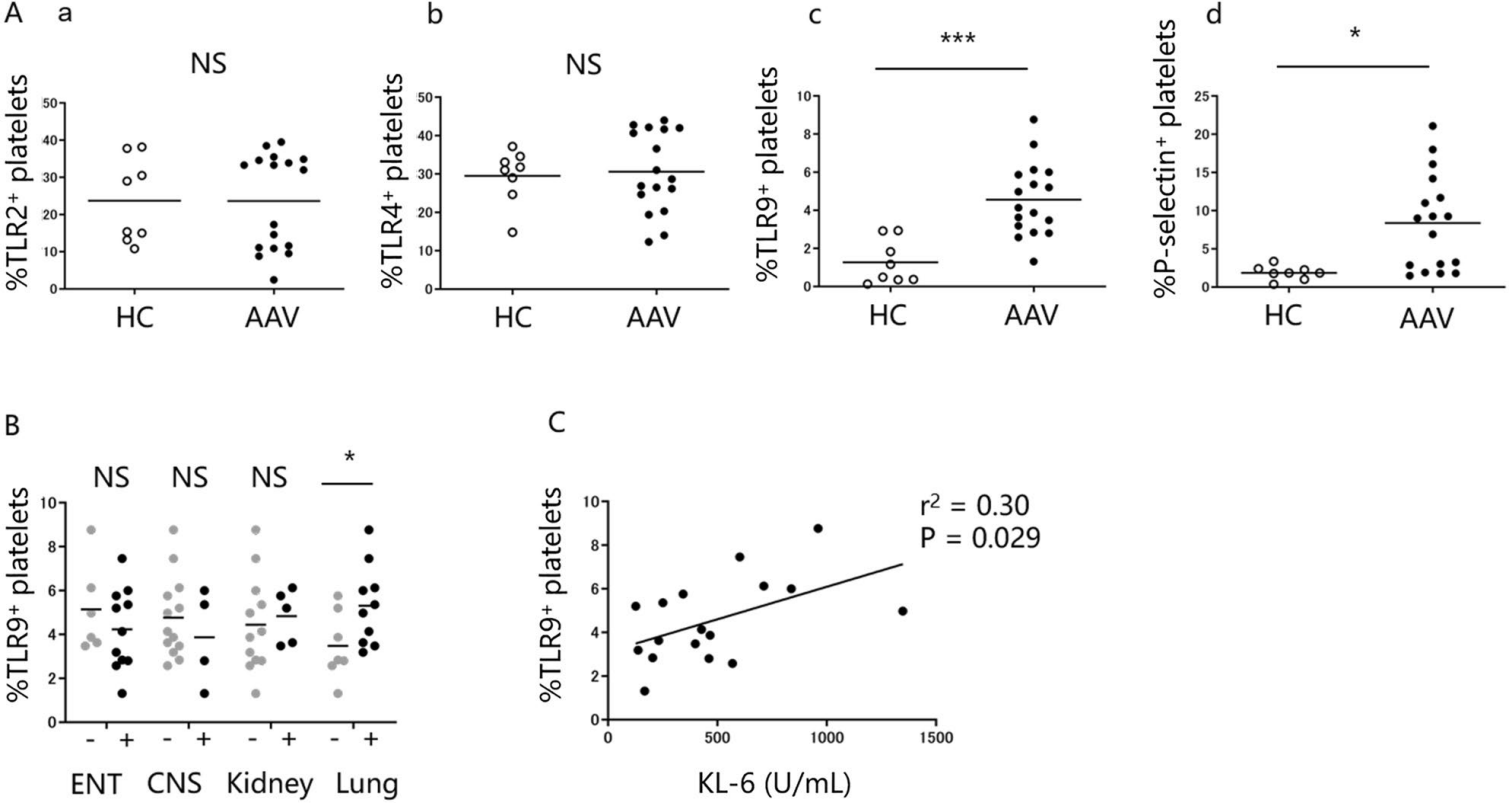
The expression level of TLR9 on platelets is associated with clinical features of patients with AAV. The expression levels of toll-like receptor (TLR) 2, 4, 9 and P-selectin on platelets from ANCA-associated vasculitis (AAV) patients (n = 17) and healthy controls (HCs) (n = 8) were analyzed by flow cytometry. **(A)** Proportion of platelets expressing TLR2 **(a)**, TLR4 **(b)**, TLR9 **(c)** and P-selectin **(d)**. **(B)** Proportion of TLR9^+^ platelets in AAV patients with or without selected organ involvement (ENT, 11:6; CNS, 4:13; kidney, 5:12; lung, 10:7). **(C)** Correlation between the proportion of TLR9^+^ platelets and serum Krebs Von Den Lungen-6 (KL-6) level in AAV patients. *p < 0.05 **p < 0.01 ***p < 0.0001 for analysis using Mann–Whitney *U* test. NS: not significant.

Various TLRs are preferentially expressed on human platelets, such as TLR2, 4 and 9^37,38^, and are involved in production of chemokines and cytokines from platelets. In addition, P-selectin is a well-known activation marker of platelets^39^. As shown in Fig. 2A, we examined the expression of platelet-associated surface molecules such as TLR2 (a), 4 (b), 9 (c) and P-selectin (d) to identify the phenotype of platelets (AAV: n = 17, HCs: n = 8). The expression levels of TLR9 and P-selectin on platelets from AAV patients was higher than HCs (1.3% vs 4.6%, p = 0.0002 and 1.9% vs 8.4%, p = 0.0071, respectively), whereas no significant difference was observed in the expression levels of TLR2 and TLR4 between AAV patients and HCs (24% vs 24%, p = 0.97 and 30% vs 31%, p = 0.92, respectively). Comparison of the proportion of TLR9^+^ platelets between patients with selected organ involvement demonstrated that the proportion was significantly higher in AAV patients with lung lesions than in those without (3.5% vs 5.3%, p = 0.043) (Fig. 2B). Moreover, the proportion of TLR9^+^ platelets was correlated with the serum KL-6 level (r^2^ = 0.27, p = 0.034) (Fig. 2C). These data suggest that elevated TLR9 expression in platelets, in particular, may contribute to the principal process of pulmonary lesions in AAV.

### Activation of TLR9 signaling pathways in platelet-induced NETs formation by CXCL4

Next, we focused on the role of TLR9 in the process of platelet-mediated NETs formation. Because platelets can store various humoral factors in granules^38^, we hypothesized that platelets can be activated by TLR9 and release humoral factors from granules to promote NETs formation. CXCL4 (platelet factor 4: PF4), a major chemokine in platelets, have been reported to activate neutrophils in vitro^40^. We examined whether TLR9 signaling could induce CXCL4 release from platelets and contribute to NETs formation using platelets from HCs.

**Figure 3:**
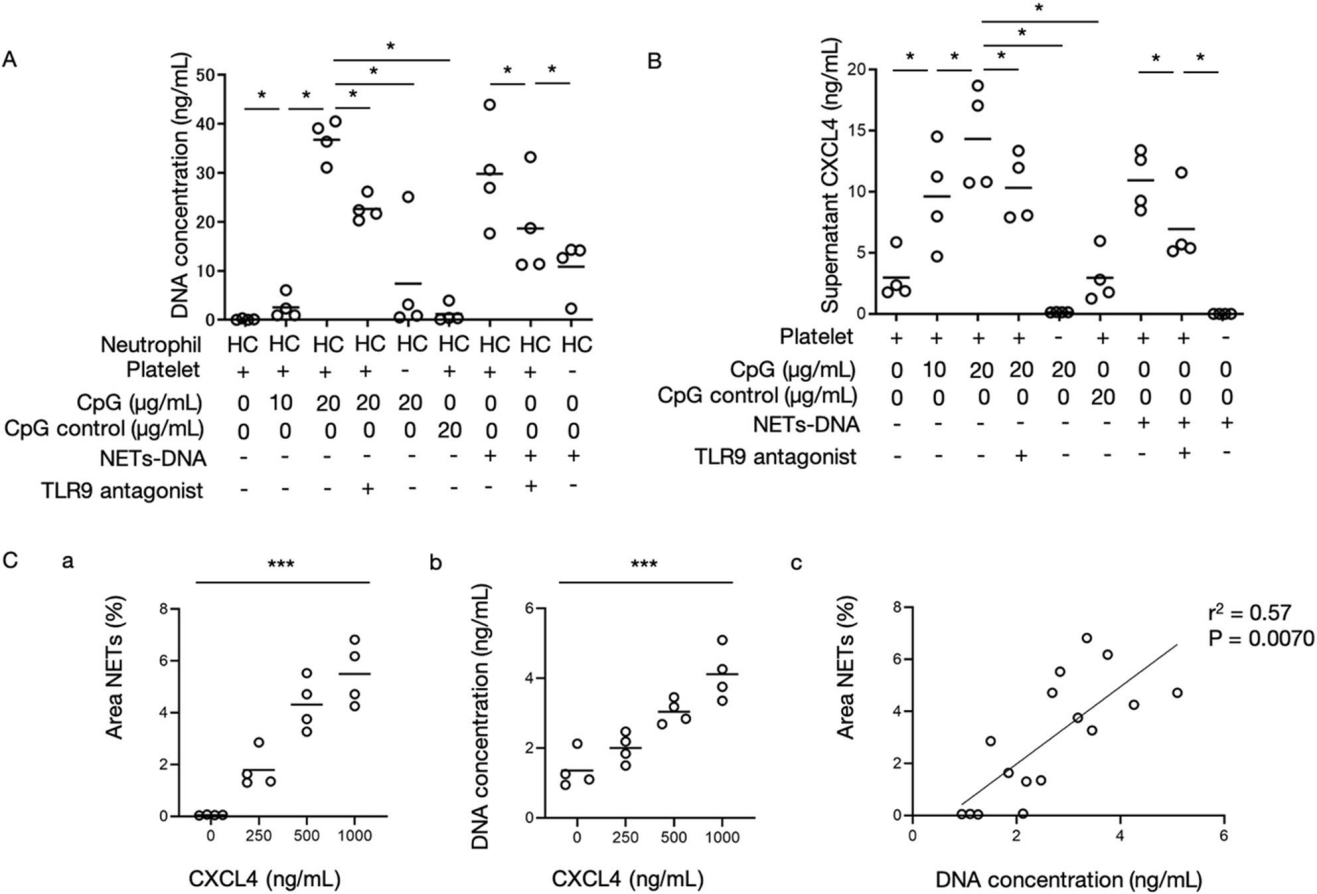
A TLR9 ligand induced CXCL4 release from platelets and NETs formation from neutrophils via CXCL4. Neutrophils and platelets from healthy controls (HCs) were cultured in the presence of CpG oligodeoxynucleotide, control nucleotide or NETs-DNA, and/or toll-like receptor (TLR) 9 antagonist. We used platelets in the platelet-rich plasma (PRP) form. Summary of 4 independent experiments using 4 different samples from HCs was shown. **(A)** Fluorescence intensity of Picogreen-labeled DNA release was measured after 1 h of incubation. **(B)** CXCL4 levels in supernatant of CpG-stimulated platelets were measured. **(C)** Neutrophil extracellular traps (NETs) formation induced by neutrophils from HCs (n = 4) in the presence of recombinant human CXCL4 was assessed by quantification of **(a)** the fluorescence intensity and **(b)** the positive signal area of Picogreen-labeled extracellular DNA. **(c)** Correlation between the fluorescence intensity and the positive signal area was shown. *p < 0.05 **p < 0.01 ***p < 0.0001 for analysis using Wilcoxon Signed-rank test **(A,B)**, Kruskal–Wallis test (**C-a**,**C-b**) and Pearson’s correlation test (**C-c**).

As shown in Fig. 3A, stimulation of platelets with CpG oligodeoxynucleotides, a TLR9 agonist, significantly enhanced NETs formation compared to stimulation with control oligodeoxynucleotides, and did so in a dose-dependent manner (0.11 vs 2.6 vs 37 ng/mL, p = 0.0002). NETs-DNA enhanced NETs formation as well. In addition, a TLR9 antagonist inhibited CpG- and NETs DNA-induced DNA release, suggesting that TLR9 signaling pathways in platelets are involved in NETs formation by neutrophils. We also examined the possible involvement of TLR9 pathways in the release of humoral factors from platelets, especially CXCL4. As shown in Fig. 3B, CXCL4 levels in culture supernatant were increased following stimulation of platelets with CpG in a dose-dependent manner (3.0 vs 9.6 vs 14 ng/mL, p = 0.026). NETs DNA-stimulated platelets enhanced CXCL4 release as well. Interestingly, CXCL4 production by CpG- and NETs DNA-stimulated platelets was suppressed by a TLR9 antagonist. These data indicate that TLR9 stimulation, in part, enhanced CXCL4 production by platelets, which may subsequently contribute to NETs formation by neutrophils.

To determine whether CXCL4 promote NETs formation, we cultured neutrophils with a recombinant CXCL4 protein. Picogreen-labeled DNA was quantified by measuring the area of label and determining fluorescence intensity. We confirmed that recombinant human CXCL4 promoted NETs formation in a dose-dependent manner (Fig. 3C-a,C-b). Further, results from the two quantification methods were correlated (Fig. 3C-c) (r^2^ = 0.57, p = 0.0070).

### TLR9-stimulation enhanced CXCL4 release by platelets and NETs formation in patients with AAV

**Figure 4:**
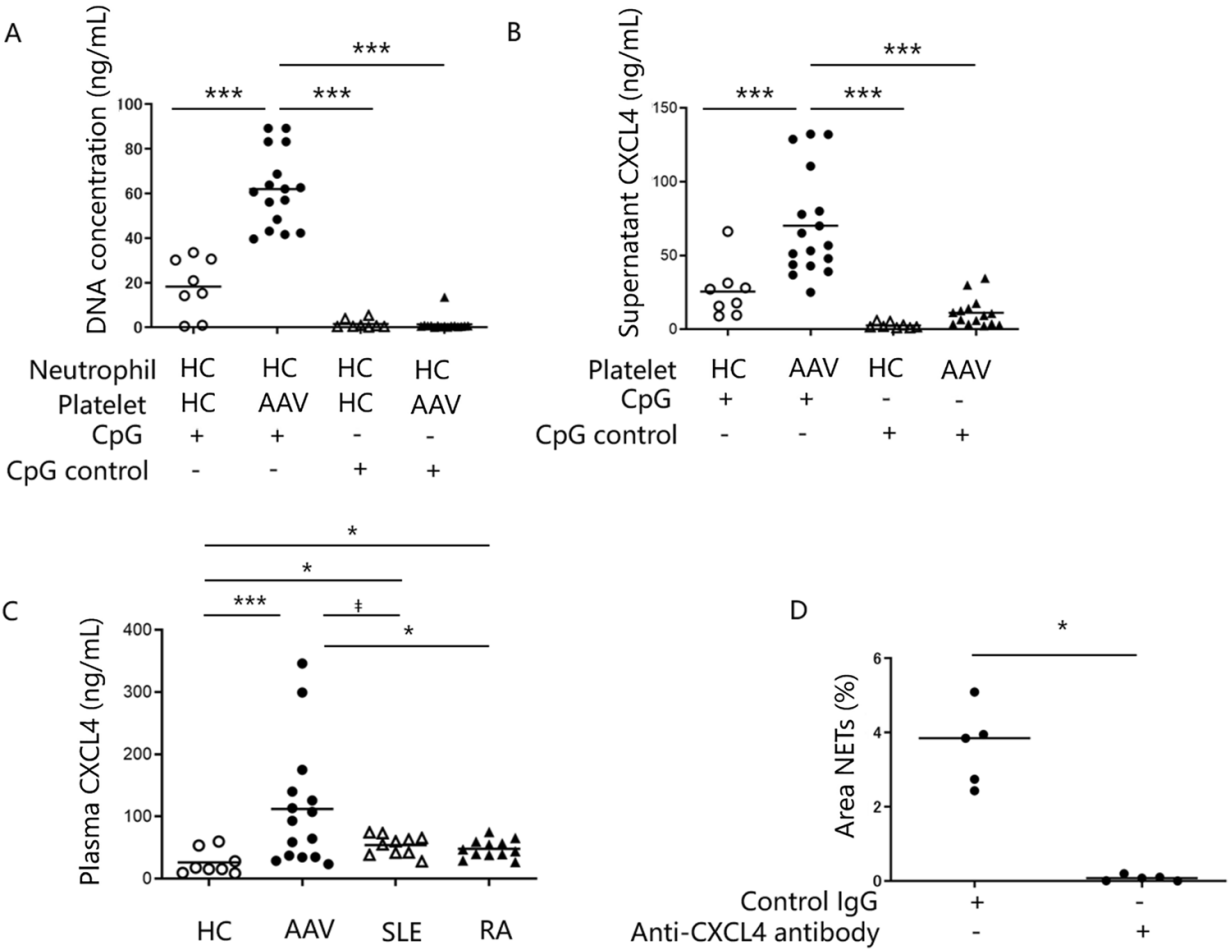
TLR9–CXCL4 signaling axis is upregulated and involved in NETs formation in AAV patients. **(A)** Neutrophils from healthy controls (HCs) were cultured with platelets from ANCA-associated vasculitis (AAV) patients (n = 17) or HCs (n = 8) treated with CpG or CpG control in an allogenic manner. Neutrophil extracellular traps (NETs) formation was analyzed by measuring the fluorescence intensity of Picogreen-labeled DNA release. We used platelets in the platelet-rich plasma (PRP) form. **(B)** The amounts of CXCL4 in the culture supernatants was measured by ELISA. **(C) **Plasma levels of CXCL4 in HCs (n = 8) and AAV (n = 17), SLE (n = 10) and RA (n = 12) patients were measured by ELISA. **(D)** NETs formation induced by PRP from AAV patients (n = 5) in the presence of anti-CXCL4 antibody or control IgG was monitored by positive signal area of Picogreen-labeled DNA. **(A–C)** p < 0.05 for Kruskal–Wallis test. ‡p < 0.1 *p < 0.05 **p < 0.01 ***p < 0.0001 for analysis using Mann–Whitney *U* test.

Next, we investigated the role of TLR9 signaling in NETs formation induced by platelets from AAV patients. As shown in Fig. 4A, the concentration of extracellularly-released DNA was higher following exposure to CpG-stimulated platelets from AAV patients than from HCs (18 vs 61 ng/mL, p < 0.0001). The amount of CXCL4 released into supernatants was also greater from CpG-stimulated platelets from AAV patients than from HCs (26 vs 70 ng/mL, p = 0.00060) (Fig. 4B), suggesting that CXCL4 released from TLR9-activated platelets may induce NETs formation in AAV.

We also measured plasma concentrations of CXCL4 in HCs and patients with AAV, SLE and RA. Plasma concentrations of CXCL4 were higher in AAV (112 ng/mL, p = 0.0010), SLE (54 ng/mL, p = 0.0060) and RA (48 ng/mL, p = 0.019) patients than in HCs (26 ng/mL) (Fig. 4C). Further, plasma concentrations of CXCL4 were higher in AAV than in SLE (p = 0.077) or RA (p = 0.033) patients. Interestingly, plasma CXCL4 concentrations correlated with level of platelet-mediated NETs shown in Fig. 1C (r^2^ = 0.62, p = 0.00050) in AAV patients (Fig. S3).

To determine whether CXCL4 have role in platelet-mediated NETs formation in AAV patients, we treated neutrophils with a neutralizing CXCL4-specific antibody in the presence of platelets from AAV patients. Representative confocal microscopy images of Picogreen-labeled extracellular DNA (Fig. S4A) and images processed using ImageJ software (Fig. S4B) were shown. CXCL4 neutralizing antibody significantly inhibited NETs formation by normal neutrophils co-cultured with platelets from AAV patients measuring the area of positive signals as described in “Methods” (3.6% vs 0.077%, p = 0.0079) (Fig. 4D). Taken together, our data suggest that the TLR9 − CXCL4 signaling axis is upregulated in AAV patients, promotes NETs formation and contributes to the pathogenesis of AAV.

## Discussion

We showed that activation of platelets through TLR signaling pathways contributes to NETs formation and the progression of AAV. We first demonstrated that platelets from AAV patients co-cultured with neutrophils induced NETs formation. We further examined the mechanisms for enhancement of NETs formation in AAV and found that phenotype of platelets of patients with AAV was altered including upregulation of TLR9 and associated with activation of platelets. Interestingly, even with platelet and neutrophils from healthy donors, both CXCL4 release from platelets and NETs formation in the co-culture system were enhanced via TLR9 stimulation. Furthermore, TLR9–CXCL4 axis is upregulated and NETs formation was also enhanced in AAV patients compared with HCs. Our data suggest that activation of TLR9 pathways in AAV platelets enhances CXCL4 release and NETs formation and contributes to the pathogenesis of AAV. In our study, platelet-mediated NETs and plasma CXCL4 were higher in AAV patients with interstitial lung disease (ILD) as compared to those without. Since patients with SLE and RA enrolled in this study did not have ILD, the involvement of platelet-mediated NETs formation in the pathogenesis of ILD in patients with SLE or RA remains to be unclear.

NETs plays a substantial role in the pathogenesis of AAV. Since NETs was observed in stimulation with PPP from patients with AAV, plasma components, such as ANCA, were considered as inducer of NETs. As Kraaij et al. reported, plasma from MPO-ANCA^+^ MPA patients induced higher levels of NETs formation^41^. Although ANCA activity is a well-known inducer of NETs, its expression is not always consistent with disease activity^41^. There is therefore a need to identify other NETs inducers. In our study, comparison of PPP and PRP suggests that platelets may be another regulator of NETs in AAV. Considering the level of platelet-mediated NETs is not associated with ANCA titer or ANCA subtypes of patients, activation of neutrophils by platelets may not be mainly mediated by ANCA-IgG, even though we do not rule out the possibility that ANCA-IgG affects platelets by stimulating Fc receptors on platelets. We demonstrated that platelets from AAV patients induced NETs formation via not only cell-to-cell contact with neutrophils but also humoral factors produced by platelets. While NETs formation in mouse models is induced by P-selectin-dependent platelet-neutrophil aggregation^42^, NETs formation in humans is not suppressed by blocking P-selectin^40^, indicating that P-selectin pathways are not involved in human platelet-induced NETs formation. However, a clinical study reported that serum levels of platelet-derived soluble proteins such as P-selectin and soluble CD40 ligand are positively correlated with AAV disease activity^43^.

TLRs, which are expressed on various immune cells such as platelets, neutrophils, lymphocytes and dendritic cells, play a central role in the detection of bacterial and viral pattern recognition, which facilitates enhanced cell-mediated inflammation^25^. TLR stimulation have been reported to induce NETs formation and to trigger the development of autoimmune diseases, including vasculitis^44^. We demonstrated here that the proportion of TLR9^+^ platelet was significantly elevated in AAV patients compared to HCs. Our data suggest that TLR9 signaling pathways in platelets are involved in NETs formation and the development of AAV. Previous reports have also shown that TLR9 is involved in the pathogenesis of AAV. For example, immunization with a TLR9 ligand enhanced MPO-ANCA-induced vasculitis and increased immune response and organ damage in a mouse model^44^. Moreover, TLR9 ligands promote ANCA production via peripheral B lymphocytes from AAV patients in vitro^45^. Given that TLR9 recognizes DNA from apoptotic cells^13^, DNA production by NETs formation may activate TLR9 on platelets, which in turn accelerates the contribution of neutrophils to NETs formation.

In addition, we revealed that stimulation of platelets with a TLR9 ligand led to the release of CXCL4, which is abundant in platelets. Our findings suggest that CXCL4 itself promotes NETs formation in a dose-dependent manner, consistent with the previous study ^40^. Thus, it is possible that TLR9 signaling leads to the release of CXCL4, which subsequently induces NETs formation. A previous report demonstrated that CXCL4 induced neutrophil- and platelet-dependent lung injury in a mouse model^46^, suggesting that CXCL4 derived from TLR9 ligand-activated platelets may contribute to lung involvement in AAV. Consistent with this, we found that the proportion of TLR9^+^ platelets was upregulated in AAV patients with lung involvement. While TLR9^+^ platelets exist in all types of tissues, platelets are especially abundant in lung tissue and activated by respiratory stimulant^47^. Regarding the stimulation of TLR9, respiratory tract infection may trigger TLR9 activity.

While our data thus far have demonstrated the role of platelets in the pathogenesis of AAV, neutrophils themselves might also contribute to the development of AAV. Several lines of evidence have demonstrated that neutrophils in AAV patients express the antigenic targets of MPO on their surface and that MPO/PR3-ANCA stimulation induces NETs formation ^48^. Another report has suggested that stimulation by TLR ligands leads to neutrophil activation and upregulation of the membrane expression of MPO/PR3^49^. Thus, stimulation of TLR9 can directly affect neutrophils and act as a primer to induce neutrophil degranulation.

Some limitations of this study warrant mention. First, because we used platelets in the PRP form, plasma components may have contributed to the observed NETs formation. Second, DNA concentration we measured may not exactly synonymous with quantification of NETs. Third, the effect of TLR9 stimulation on platelets is greater than expected based on the relatively small difference in TLR9 expression. The results of genome-wide association studies suggest that polymorphisms of TLR9 are associated with the development of AAV^50^. SNPs, in addition to expression level, may be involved in TLR9–CXCL4 axis, although further study is needed to confirm this. Fourth, our sample size was too small to conduct comparisons between patients in the active and remission phases. At last, platelet-mediated signals such as DAMP or HMGB1 were not described in this study. Confirmation of our findings awaits further investigation.

**Figure 5:**
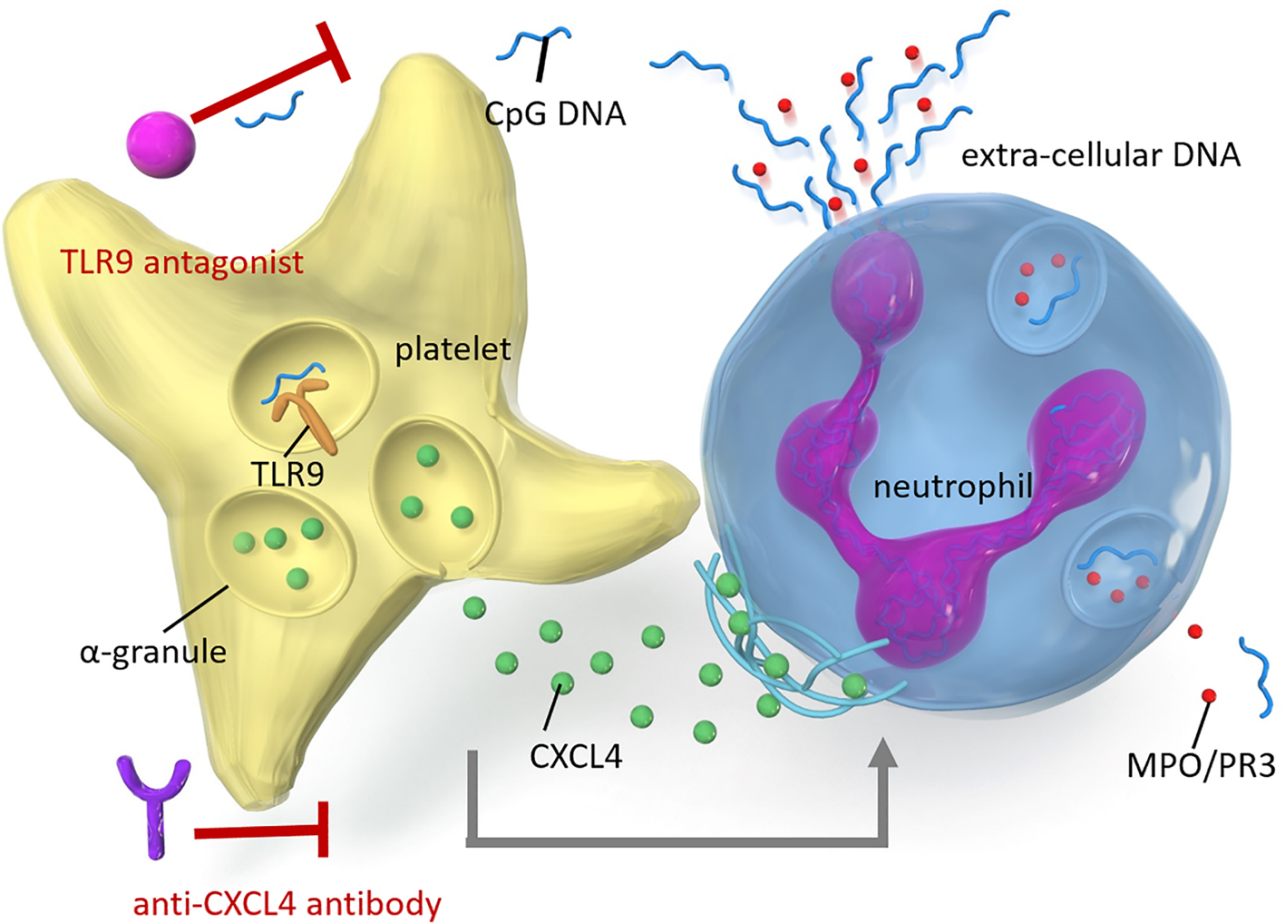
Schematic illustration of the proposed mechanism of platelet-mediated NETs formation in AAV. CXCL4 released from TLR9 agonist-stimulated platelets from AAV patients markedly increased NETs formation. TLR9 signaling and CXCL4 release are essential mediators of NETs formation in AAV, with the effects of these mediators being inhibited by a TLR9 antagonist or anti-CXCL4 antibody.

In conclusion, our findings suggest that activation of platelets through TLR9 signaling pathways contributes to NETs formation by releasing CXCL4 and the progression of AAV. We demonstrated that the TLR9–CXCL4 signaling axis was upregulated and NETs formation was enhanced in AAV patients compared with HCs. Moreover, we found that activation of TLR9 pathways in AAV platelets enhances CXCL4 release and NETs formation. As shown in Fig. 5, we hypothesize that enhanced TLR9–CXCL4 axis results in inducing NETs formation and is involved in the pathogenesis of AAV. Further evidence on the mechanisms of the crosstalk between platelets and neutrophils will aid in the identification of novel therapeutic targets for the treatment of AAV.

## Supplementary Information

Supplementary Information.
