# Supplementary material for: Platelet CXCL4 mediates neutrophil extracellular traps formation in ANCA-associated vasculitis
Source: Sci Rep. 2021 Jan 8;11:222. doi: 10.1038/s41598-020-80685-4 (PMC7794527; doi:10.1038/s41598-020-80685-4)
Supplement: Supplementary file 1 — Supplementary Information. [file 41598_2020_80685_MOESM1_ESM.docx]

**ORIGINAL RESEARCH**

**Platelet CXCL4 mediates neutrophil extracellular traps formation in ANCA-associated vasculitis**

Kotaro Matsumoto^1^, Hidekata Yasuoka^1,2^, Keiko Yoshimoto^1^, Katsuya Suzuki^1^, Tsutomu Takeuchi^1*^

^1^ Division of Rheumatology, Department of Internal Medicine, Keio University School of Medicine, 35 Shinanomachi, Shinjuku-ku, Tokyo, 160-8582, Japan

^2^ Division of Rheumatology, Department of Internal Medicine, Fujita Health University School of Medicine, 1-98 Dengakugakubo, Kutsukake-Cho, Toyoake, Aichi, 470-1192, Japan

Correspondence should be addressed to: Tsutomu Takeuchi, Division of Rheumatology, Department of Internal Medicine, Keio University School of Medicine, 35 Shinanomachi, Shinjuku-ku, Tokyo, 160-8582, Japan. E-mail: tsutake@z5.keio.jp, Phone: +81-3-5363-3786, Fax: +81-3-5379-5037

Table S1. Clinical characteristics of patients with SLE and RA, and HCs

| Parameter | SLE (n=10) | RA (n=12) | HC (n=20) |
| --- | --- | --- | --- |
| **Baseline characteristics** |  |  |  |
| Age, years | 47±14 | 57±16 | 49±15 |
| Male, n (%) | 2 (20) | 3 (25) | 9 (45) |
| SLEDAI | 8.2±6.9 | - | - |
| Stage: I/II/III/IⅤ, n | - | 3/7/1/1 | - |
| Class: I/II/III/IⅤ, n | - | 1/8/1/2 | - |
| SDAI | - | 20±12 | - |
| Newly diagnosed/Under treatment, n (%) | 9 (90)/1 (10) | 7 (58)/5 (42) | - |
| **Characteristics of patients under treatment** |  |  |  |
| Disease duration, months | 64 | 62±71 | - |
| Treatment, n | PSL, 1 | PSL, 1; MTX, 4 | - |
| **Laboratory tests** |  |  |  |
| WBC, cells/μL | 4,570±1,286 | 6,500±2,019 | - |
| Neutrophils, cells/μL | 3,482±985 | 4,429±1751 | - |
| Lymphocytes, cells/μL | 808±488 | 1,489±439 | - |
| Monocytes, cells/μL | 197±93 | 391±107 | - |
| Eosinophils, cells/μL | 59±71 | 150±147 | - |
| Basophils, cells/μL | 6.9±9.9 | 40±23 | - |
| Platelets, ×10^4^ cells/μL | 18±10 | 26±7.2 | - |
| Creatinine, mg/dL | 0.7±0.3 | 0.6±0.2 | - |
| eGFR, mL/min/1.73m^2^ | 85±21 | 87±23 | - |
| CRP, mg/dL | 0.5±0.7 | 0.8±0.5 | - |
| Anti-ds DNA antibody positive, n (%) | 8 (80) | - | - |
| Anti-CCP antibody positive, n (%) | - | 10 (83) | - |
| Rheumatoid factor positive, n (%) | 2 (20) | 10 (83) | - |
| **Comorbidities** |  |  |  |
| Interstitial lung disease, n (%) | 0 (0) | 0 (0) | - |
| Sjögren's syndrome, n (%) | 3 (30) | 0 (0) | - |
| Antiphospholipid antibody syndrome, n (%) | 2 (20) | 0 (0) | - |

SDAI: simplified disease activity index, PSL: prednisolone, MTX: methotrexate, WBC: white blood cell, ds DNA: double stranded DNA

Table S2. Levels of PRP- and PPP-induced NETs formation in HCs and patients with AAV, SLE and RA.

| DNA concentration | HC, n=20 | | |
| --- | --- | --- | --- |
| PRP, ng/mL | 3.2±3.4 | | |
| PPP, ng/mL | 3.2±3.7 | | |
| $\Delta$PRP−PPP, ng/mL | 0.0003±1.2 | | |
|  | AAV | | |
|  | Total, n=22 | Newly diagnosed, n=18 | Under treatment, n=4 |
| PRP, ng/mL | 23.8±62.1 | 28.0±68.2 | 4.9±4.9 |
| PPP, ng/mL | 18.7±52.9 | 21.9±58.2 | 4.5±4.8 |
| $\Delta$PRP−PPP, ng/mL | 5.4±10.7 | 6.1±11.7 | 2.3±3.6 |
|  | SLE | | |
|  | Total, n=10 | Newly diagnosed, n=9 | Under treatment, n=1 |
| PRP, ng/mL | 4.3±5.4 | 4.7±5.5 | 0.1 |
| PPP, ng/mL | 5.1±5.3 | 5.7±5.3 | 0.2 |
| $\Delta$PRP−PPP, ng/mL | -0.9±2.8 | -1.0±2.9 | -0.05 |
|  | RA | | |
|  | Total, n=12 | Newly diagnosed, n=7 | Under treatment, n=5 |
| PRP, ng/mL | 5.3±6.0 | 8.8±5.6 | 0.5±0.2 |
| PPP, ng/mL | 5.7±6.4 | 9.4±5.9 | 0.5±0.2 |
| $\Delta$PRP−PPP, ng/mL | -0.4±1.7 | -0.6±2.2 | -0.02±0.1 |

PRP: platelet-rich plasma, PPP: platelet-poor plasma, HC: healthy control, AAV: ANCA-associated vasculitis, SLE: systemic lupus erythematosus, RA: rheumatoid arthritis

**Supplementary Figure legends**

Fig. S1. Details of the gating strategies of platelets.


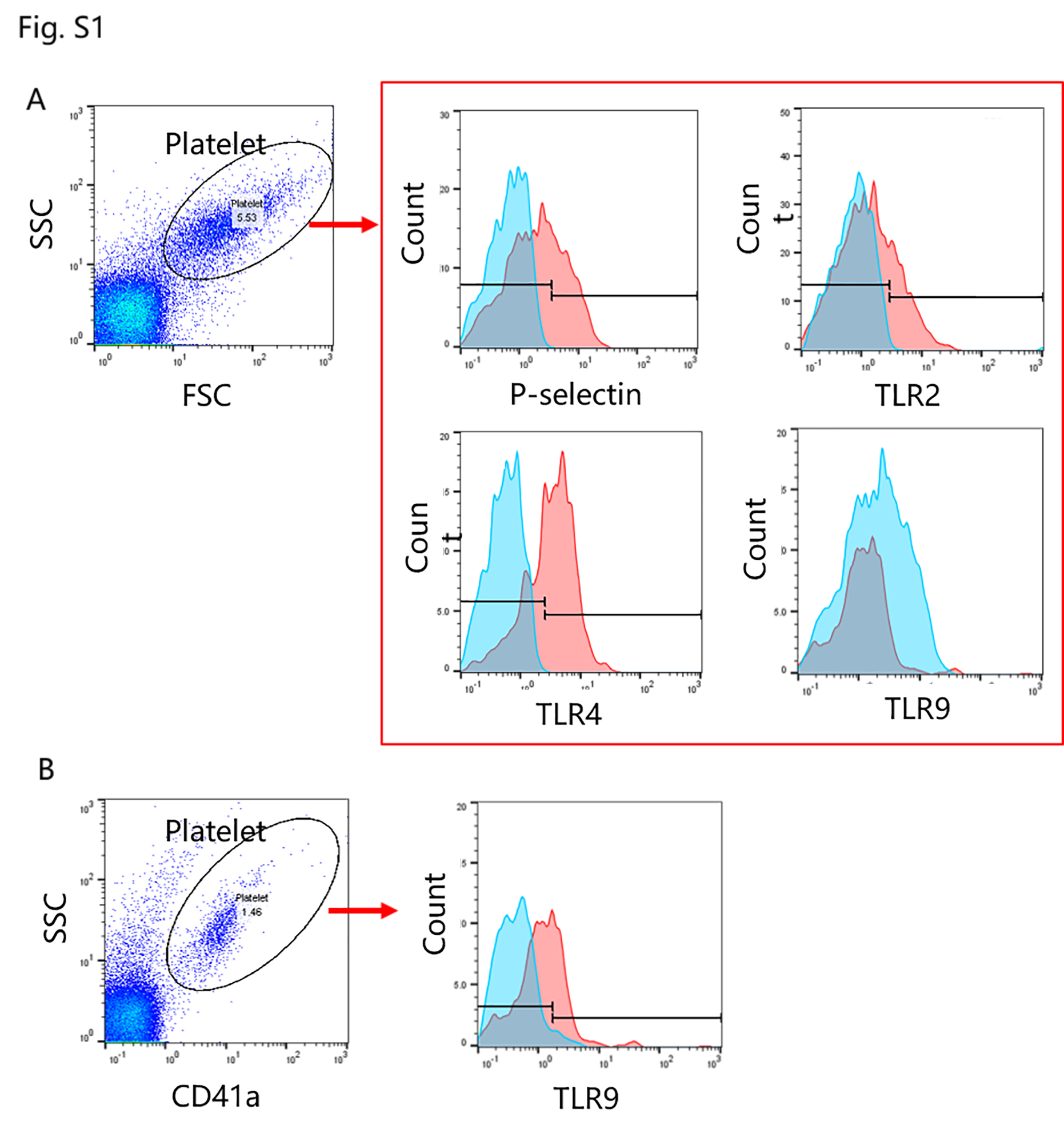


Gating strategy for (A) surface molecules, P-selectin, toll-like receptor (TLR) 2, 4 and 9, and (B) intracellular TLR9 after permeabilization of the platelets. Blue: isotype-matched control IgG.

Fig. S2. Platelets from AAV patients enhanced NETs formation.

(A) Representative images of NETs formation showing positive signal area of Picogreen labeled DNA. Neutrophils from HCs (a) and AAV (b) without treatment with platelet-poor plasma (PPP) or platelet-rich plasma (PRP). Neutrophils from HC pretreated with PPP from HCs (c), PRP from HCs (d), PPP from AAV (e) and PRP from AAV (f). PPP from AAV (g) and PRP from AAV (h) without neutrophils. Neutrophils from HCs pretreated with PRP from AAV patients in a trans-well assay that prevented passage of cells of size 0.4 μm or greater (i). Scale bar: 100 μm. The experiment was repeated four times. (B) Concentration of DNA released following exposure of normal neutrophils to platelet rich plasma or platelet poor plasma from HCs (n = 20) and AAV (n = 22), SLE (n = 10) and RA (n = 12) patients. *p<0.05 **p<0.01 ***p<0.001 for analysis using Mann-Whitney *U* test.

Fig. S3. Correlation between platelet-mediated NETs and plasma CXCL4.


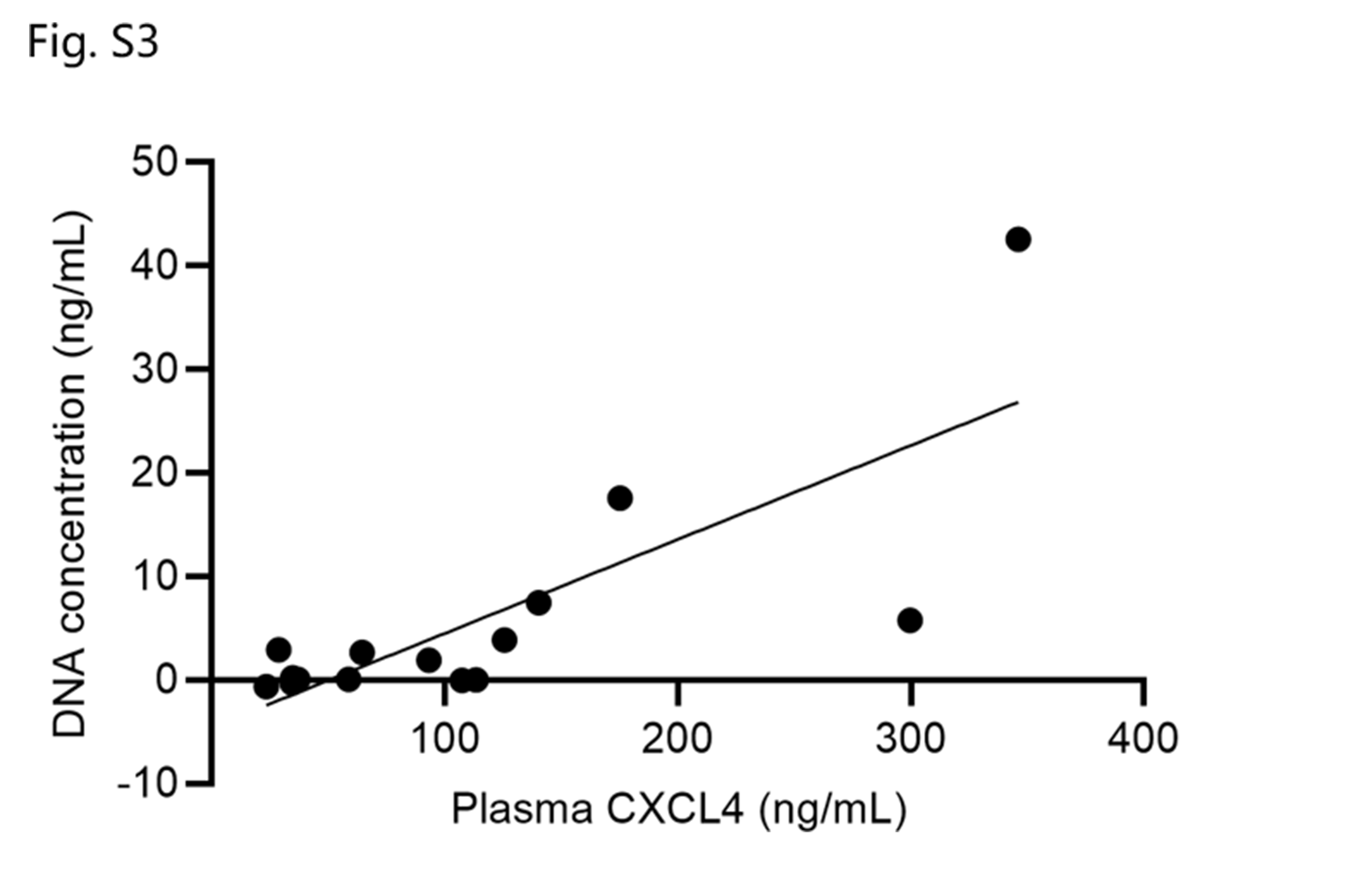


Levels of platelet-mediated NETs (shown in Fig. 1C) correlated with levels of plasma CXCL4 concentration in AAV patients (n=17).

Fig. S4. Anti-CXCL4 antibody inhibited platelet-mediated NETs formation.


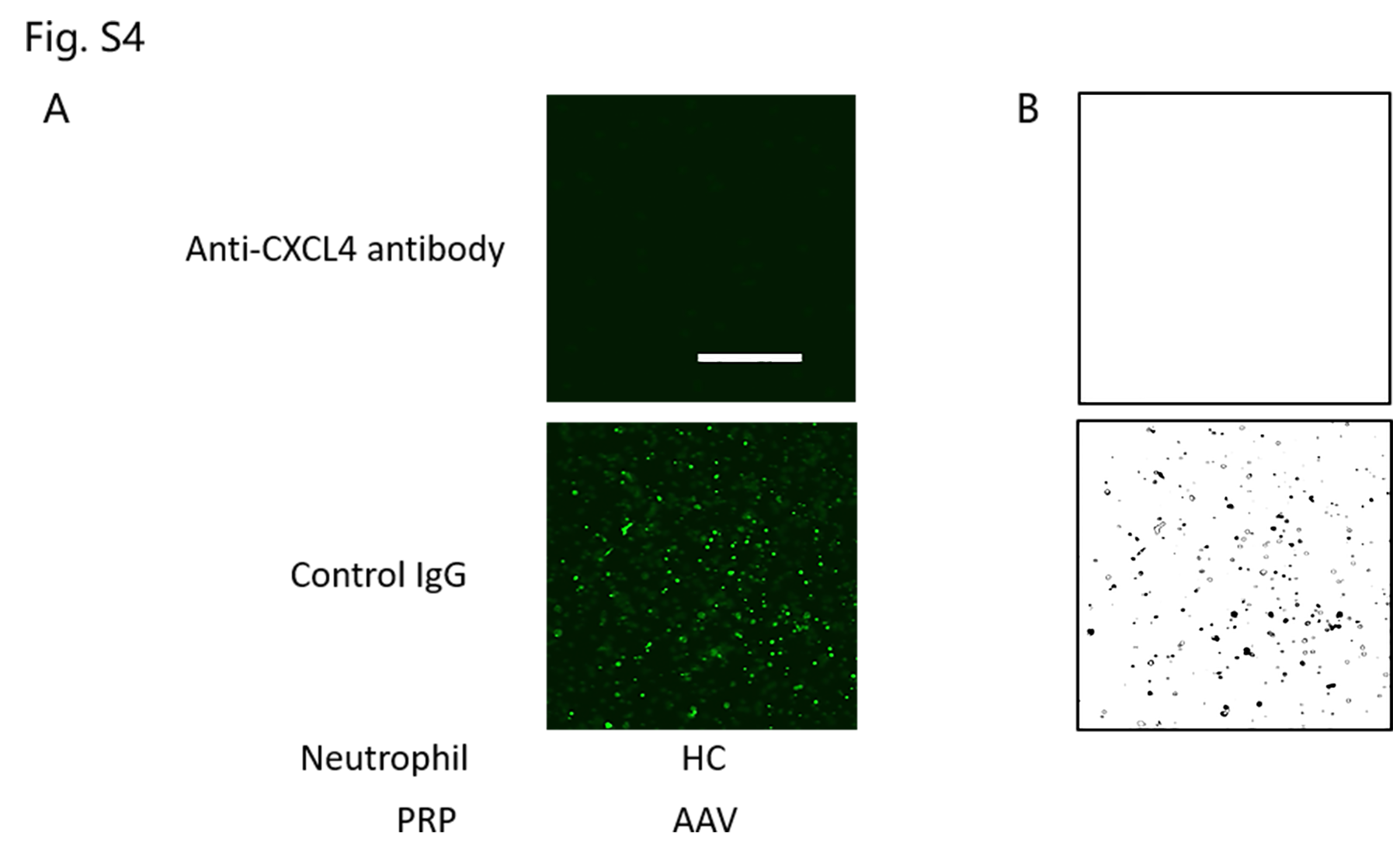


Representative images of neutrophil extracellular traps (NETs) formation by neutrophils from healthy controls (HCs) in the presence of anti-CXCL4 antibody or control IgG. (A) confocal microscopy images and (B) images processed using ImageJ software. Scale bar: 100 μm.
